# Performance of Older Adults on the Digital Clock and Recall Test Compared to the Montreal Cognitive Assessment in Primary Care Settings

**DOI:** 10.1007/s11606-025-09702-4

**Published:** 2025-07-09

**Authors:** Dustin B. Hammers, Daniel Schulman, Nicole R. Fowler, Jane Musema, Jared R. Brosch, Diana Summanwar, Kristen Swartzell, Connor Higgins, Russell Banks, Katherine J. Selzler, Timothy MacLeod, Sean Tobyne, Deanna R. Willis

**Affiliations:** 1 Department of Neurology, Indiana University School of Medicine, 355 West 16th Street (GH4027), Indianapolis, IN 46202 USA; 2 Linus Health, Boston, MA USA; 3 Department of Medicine and IU Center for Aging Research, Indiana University School of Medicine, Indianapolis, IN USA; 4 Department of Family Medicine, Indiana University School of Medicine, Indianapolis, IN USA; 5 Purdue University School of Nursing, West Lafayette, IN USA; 6 Michigan State University, East Lansing, MI USA; 7 Davos Alzheimer Collaborative, Wayne, PA USA

**Keywords:** Cognition, Digital cognitive assessments, Dementia, Primary care

## Abstract

**Background:**

Digital cognitive assessment solutions can overcome some barriers to cognitive screening in primary care by providing rapidly obtained objective insights without requiring specialty trained examiners. Real-world comparison of digital assessments to standard screenings in primary care is limited.

**Objective:**

Our objective was to compare performance on the Linus Health Digital Clock and Recall (DCR™) to the Montreal Cognitive Assessment (MOCA), which is a traditional “gold standard” cognitive screening test in primary care.

**Participants:**

A total of 114 primary care patients ≥ 65 years old completed a DCR as part of routine primary care and scored in the “*Borderline*” or “*Impaired*” ranges, and subsequently completed a MOCA at a follow-up primary care visit.

**Design:**

Criterion and convergent validity analyses were conducted using Mann–Whitney *U* tests, concordance (agreement) rates, polychoric or polyserial correlations, and exploratory factor analysis.

**Main Measures:**

DCR and MOCA Total Scores and subcomponent scores.

**Key Results:**

DCR Total Score and select process scores successfully discriminated impairment on the MOCA using traditional cutoffs, and both agreement rates and correlations were strong between DCR and MOCA components—especially Total Scores comparisons. Exploratory factor analysis revealed a five-factor model whereby one factor was comprised of memory subtests from both the DCR and MOCA, and another was comprised of non-memory MOCA subtests and Information Processing/Spatial Reasoning subcomponents of the DCR.

**Conclusions:**

Screening in primary care using the DCR is feasible, and shows criterion and convergent validity with a “gold standard” screening tool for detecting cognitive impairment—the MOCA. When paired with parallel advancements in detection of plasma-based biomarkers and recent FDA approvals for disease-modifying treatments for Alzheimer’s disease, the DCR and similar digital cognitive assessment tools have the potential to triage patients for both Alzheimer’s disease diagnostic workups and subsequent treatments beyond specialty care.

## INTRODUCTION

Recent advancements in the detection of and treatment of Alzheimer’s disease (AD) highlight the need for rapid detection of cognitive impairments to intervene early in the disease process. As many patients reach specialty cognitive neurology clinics with cognitive impairments too severe for current AD treatments, primary care is the optimal setting to target those potentially eligible for treatment. Additional screening tools available in a primary care setting could therefore lead to earlier recognition of dementia. Digital cognitive screening solutions can overcome some barriers to paper and pencil cognitive assessments in primary care settings by providing rapidly obtained objective insights without requiring specialty-trained examiners for administration, recording, and scoring of examinee’s responses.^1^ Digital screening tools are more temporally and financially efficient than standard neuropsychological batteries,^2^ and are also less susceptible to practice effects upon repeat administration due to the ability to offer alternative “forms” of stimuli.^3^

While adoption of digital technology in healthcare has expanded dramatically in recent years,^4^ acceptance of specific digital cognitive screening tools is dependent on belief in their construct validity (“does it measure what it says?”) and external validation.^5^ The Linus Health Digital Clock and Recall (DCR™; https://www.linushealth.com) is a three-part test consisting of three-word immediate verbal acquisition, the Digital Clock Test (DCTclock™), and delayed recall of the three words. The DCR was designed as the digital, process-driven evolution to the Mini-Cog^6^ that has been tested in primary care. The DCR is tablet-based and incorporates process-related scoring algorithms to identify subtle abnormalities more sensitively in a variety of cognitive domains, including verbal memory, attention and executive function, visuospatial skills, receptive and expressive language, and simple and complex motor skills. Recent work has separately shown that the DCR performs better than the Mini-Cog and the Mini-Mental State Examination (MMSE)^7^ in discriminating clinical trial participants with Mild Cognitive Impairment (MCI) or early dementia due to AD from those who are cognitively intact.^8,9^ Additionally, lower scores on the DCTclock component of the DCR have been associated with increased risk of a diagnosis of dementia in African American populations within 3–5 years of cognitive screening.^10^ Although these results are encouraging, some skepticism about the usefulness of the DCR may remain given the myriad other cognitive screening tools available in many settings. Consequently, the principal objective of this study was to compare performance on the DCR to the Montreal Cognitive Assessment (MOCA), which is one of the most widely known and commonly used cognitive screening tests in primary care.^11,12^ Our main hypothesis is that there will be strong criterion and convergent validity for the DCR relative to MOCA for routine screening in primary care. Such results would advance evidence of validity for the DCR and increase confidence in its use by primary care providers.

## METHODS

### Participants

Adults ≥ 65 years old presenting for any issue to one of seven diverse primary care clinics between 06/2022 and 05/2023 were asked to complete the Linus Health DCR as part of a larger implementation study (*the Davos Alzheimer’s Collaborative Healthcare System Preparedness Early Detection Flagship Program*) to understand the barriers and facilitators to implementing digital cognitive assessments in primary care.^13^ Each clinic was chosen to represent suburban and urban locations, and a racially and socioeconomically diverse sample, within a statewide academic health care system. At the discretion of their primary care provider, patients who scored abnormally on the DCR (as described below) were offered a clinical follow-up visit with a primary care–based Brain-Health Navigator (BHN), at which point they were administered the MOCA as part of the BHN protocol. Overall, 114 individuals screened abnormally on the DCR and agreed to a follow-up BHN visit where they received a MOCA. The sample was on average 73.9 years old, with a mean time of 65.0 days between DCR and MOCA evaluations (range 0–224 days). Inclusion criteria for the implementation study involved being ≥ 65 at the time of DCR screening, fluent in English or Spanish, and an established or new primary care patient. Relevant exclusion criteria included having an existing diagnosis of dementia documented in medical records based on ICD-10 codes, and sensory impairment (e.g., visual or hearing loss) or other issues precluding the completion of DCR screening. Exclusion criteria for the current project also included having an unimpaired score on DCR, and declining follow-up with the BHN. Institutional Review Board approval was provided by Indiana University, with a waiver of written consent obtained.

### Procedure

All participants in the current study received both the DCR and MOCA, with scoring as follows:

The Linus Health DCR™ is an FDA Class II medical device, and is a digital and artificial intelligence (AI)–enabled evolution of the paper-based Mini-Cog assessment. The tablet-based DCR detects signs of cognitive impairment by analyzing the individual’s performance using a combination of digital clock drawing test (DCTclock) and 3-word immediate and delayed verbal recall tests. The total score on the DCR ranges from 0 to 5, with scores of 0–1 categorized as *indicative of cognitive impairment* (“*Impaired*” hereafter), 2–3 as *borderline for cognitive impairment* (“*Borderline*” hereafter), and 4–5 as *not indicative of cognitive impairment* (“*Unimpaired*” hereafter). Consistent with use of this tool to screen for any form of cognitive impairment, impairment cutoffs were currently set at DCR Total Score ≤ 3. The delayed recall subtest contributes 0–3 points to the total score (based on the number of words correctly recalled). The DCTclock subtest contributes 0–2 points, based on a transformed summary score ranging 0–100 with cutoff scores of < 60, 60–74, and ≥ 75 contributing 0–2 points, respectively. The DCTclock includes four Command and Copy Clock composite scales (each combining a number of lower-level features) evaluating different aspects of the clock drawing process: simple and complex motor skills, drawing efficiency, spatial reasoning, and information processing speed.^14^

The MOCA is a pencil-and-paper-based cognitive screening tool comprised of eight indexes, including visuospatial/executive skills, animal naming, attention, language, verbal abstraction, delayed recall of five unrelated words, and orientation. Scores range from 0 to 30, with higher scores indicating better performance. For the current project, MOCAs were administered by a primary care team member having completed relevant training, and cutoffs for impairment were set at MOCA total score < 26 based on developer’s recommendations.^11^

### Primary Data Analysis

Descriptive statistics were first implemented to characterize demographic and MOCA scores stratified by DCR performance, using Welch’s *t *tests for continuous variables and Fisher’s exact tests for categorical variables. Criterion validity then was assessed using Mann–Whitney* U* tests between several DCR components for participants performing above and below the MOCA cutoff. Significance was estimated by permutation tests, and measures of effect size were expressed as Vargha and Delaney’s* A* with comparisons between *A* estimates using 95% confidence intervals (CIs) estimated by nonparametric bootstrap.

To assess convergent validity, agreement rates between participant impairment on the MOCA Total score, the MOCA Recall subscore (0–5), and DCR were calculated as a proportion: the number of times a set of classifications were the same, divided by the total number of observations. Additionally, following FDA guidelines,^15^ positive and negative agreement rates were reported. Positive agreement is the agreement rate for participants identified as impaired by the DCR, and negative agreement is the agreement rate for participants identified as not impaired by the DCR. We estimated 95% CIs on all agreement rates using the Agresti-Coull method.^16^

Polychoric or polyserial correlation coefficients (as appropriate) were estimated between DCR and MOCA scores/subscores to further assess convergent validity. Polychoric correlation and polyserial correlation treat ordinal variables as if they have been made discrete using underlying normally distributed latent variables. Simulation studies have shown that the use of these methods in exploratory factor analysis can yield solutions that more accurately reproduce the true measurement model.^17^ DCTclock score was treated as continuous, and all others treated as ordinal. Two-stage estimation was used, with CIs and statistical significance estimated by nonparametric bootstrap.

Alpha levels were set at 0.05 for all analyses. Given the preliminary nature of these findings, no corrections for multiple comparisons were implemented.

### Exploratory Data Analysis

Exploratory data analysis examined whether the DCR and MOCA measure the same domains of cognitive functioning. First, Mann–Whitney* U* tests were undertaken to compare performance on the eight composite subscales of the DCTclock score between participants performing above and below MOCA cutoffs. Significance and effect sizes were estimated as above. Additionally, exploratory factor analysis was performed based on the correlation matrix of polychoric/polyserial correlations between DCR and MOCA subscores. To choose the number of factors, we first applied parallel analysis, which is a widely used method that compares the eigenvalues of the correlation matrix to those of randomly generated correlation matrices.^18^ The results of parallel analysis were used to identify a plausible range, with a final choice by consensus and based on the interpretability of the latent factors. Maximum likelihood estimation and varimax rotation were used in all cases.

Maximum likelihood estimation was used in all cases.

## RESULTS

### Demographics and MOCA Performance

Table 1 displays demographic characteristics and MOCA performances of the 114 participants in the study, as a function of performance on the DCR. Sixty percent of participants had some degree of difficulty on the DCR and were classified as *Borderline*, and 40% were classified as *Impaired*. Participants in the *Impaired* group were significantly older than those in the *Borderline* group (*t*(59.3) = 2.59, *p* = 0.012, Cohen’s* d*s = 0.55). A similar percentage of participants were female between groups (*p* = 0.846), and no differences in racial/ethnic composition (*p* = 0.566) nor educational attainment (*p* = 0.942) were observed. The mean MOCA Total Score was lower in the *Impaired* group (*t*(112) = 3.50, *p*s < 0.001, Cohen’s* d*s = 0.67).

**Table 1:** Demographics and MOCA Total Score Stratified by DCR Score

	Borderline impaired (DCR 2–3)		Impaired (DCR 0–1)	
Variable	Value	Range	Value	Range
*N*	63		42	
Age (years)	72.2 (5.4)	59–88	76.4 (9.4)	44–92
Education (%)				
< High school graduate	3.4%		2.6%	
High school graduate	37.9%		38.5%	
Some college	31.0%		33.3%	
College graduate	12.1%		15.4%	
Beyond college	15.5%		10.3%	
Sex (female, %)	54.4%		58.7%	
Race (%)				
Caucasian	51.5%		47.8%	
Black/African American	35.3%		41.3%	
Asian	2.9%		4.3%	
Ethnicity (% Hispanic/Latino)	17.6%		6.5%	
MOCA Total Score	23.65 (3.3)	13–29	21.20 (4.2)	10–28

*DCR*, Linus Health Digital Clock and Recall; *MOCA* Montreal Cognitive Assessment. Values for Age and MOCA Total Score reflect *Mean* (*SD*); all others reflect the percent of the designated demographic group

### Primary Data Analyses

Table 2 compares selected DCR scores between groups stratified by MOCA Total Score impairment cutoff (Total Score < 26). Participants performing below MOCA impairment cutoffs had significantly lower DCR Total Scores than those performing above cutoffs (*p* = 0.02, *A* = 0.643). The same result was seen for multiple variables related to the DCTclock, including the DCTclock Process Summary Score, the DCTclock Categorized Score (which is scored comparable to the Mini-Cog), and the DCTclock Command Clock scored on MOCA scale (*p*s = 0.008–0.49, *A*s = 0.595–0.673)—the latter of which is command portion of the clock when using the traditional MOCA scoring algorithm. No differences were observed for the DCR Delayed Recall Score or the DCTclock Command Clock scored on MOCA scale (*p*s = 0.059–0.531, *A*s = 0.538–0.603)—the latter of which is the copy portion of the clock using MOCA scoring.

**Table 2:** Mean Ranks of DCR Performance, Stratified by MOCA Impairment

DCR variable	MOCA unimpaired (≥ 26)	MOCA impaired (< 26)	*U*	*p*	*A*	95% CI
DCR Total Score (0–5)	3.0 (2.0)	2.0 (2.0)	838.5	0.021	0.643	0.512–0.753
DCTclock Process Summary Score (0–100)	56.0 (17.0)	45.0 (25.5)	767.0	0.008	0.673	0.547–0.780
DCTclock Categorized Score (0–2)	0.0 (1.0)	0.0 (0.0)	950.5	0.049	0.595	0.504–0.701
DCR Delayed Recall Score (0–3)	1.0 (2.0)	2.0 (1.0)	1084.5	0.531	0.538	0.419–0.655
DCTclock Command Clock scored on MOCA scale (0–3)	3.0 (1.0)	2.0 (1.0)	843.0	0.010	0.641	0.540–0.724
DCTclock Copy Clock scored on MOCA scale (0–3)	3.0 (0.0)	3.0 (1.0)	933.5	0.059	0.603	0.499–0.676

*MOCA*, Montreal Cognitive Assessment; *CI*, confidence interval; *DCR*, Linus Health Digital Clock and Recall. For all variables, the DCTclock reflects the digital clock drawing portion of the DCR, and the parenthetical values in the left column represent the ranges of possible scores for each DCTclock variable. For the lower two rows, “DCTclock Command/Copy Clock scored on MOCA scale (0–3)” represents the process score values when using the MOCA scoring system for the clock. MOCA impairment cutoff set at Total Score < 26. Statistics represent the Mann–Whitney* U* and *p* values when comparing mean ranks of variables between groups stratified by MOCA impairment, along with associated Vargha and Delaney’s* A* effect sizes and 95% CIs. Following Vargha and Delaney,^28^ *A* values from 0.56 to 0.64 are interpreted as small, 0.64 to 0.71 as medium, and 0.71 or greater as large

Table 3 shows concordance between DCR and MOCA for several impairment cutoffs and conditions. Good overall agreement (0.72) was seen between standard DCR and MOCA Total Score impairment cutoffs; as only participants with DCR Total Scores ≤ 3 received MOCA testing in the implementation project, negative agreement is not possible and positive/overall agreement was synonymous. When considering more stringent DCR impairment cutoffs that require missing items on delayed recall, positive agreement was good with both the standard MOCA Total Score cutoff (0.72) and one involving items missed on delayed recall of the MOCA (0.81). However, negative agreement in these scenarios was low (0.30 and 0.19, respectively). Consequently, participants considered impaired by this DCR diagnostic condition were likely to also be considered impaired by these MOCA cutoffs, but participants considered unimpaired are unlikely to be considered unimpaired by MOCA.

**Table 3:** Rates of Agreement Between DCR and MOCA Across Several Conditions and Cutoffs

		Agreement rates		
DCR condition	MOCA cutoff	Overall (95% CI)	Positive (95% CI)	Negative (95% CI)
Total 0–3	Total < 26	0.72 (0.65–0.78)	–	–
Total 0–3 and DCR Recall 0–2	Total < 26	0.63 (0.55–0.70)	0.72 (0.64–0.79)	0.30 (0.18–0.46)
Total 0–3 and DCR Recall 0–2	MOCA Recall < 5	0.67 (0.60–0.74)	0.81 (0.73–0.87)	0.19 (0.09–0.34)

*DCR*, Linus Health Digital Clock and Recall; *MOCA*, Montreal Cognitive Assessment; *CI*, confidence interval; *DCR Recall*, delayed recall portion of the DCR; *MOCA Recall*, delayed recall portion of the MOCA. MOCA impairment cutoff set at Total Score < 26. DCR conditions were as follows: DCR Condition “Total 0–3” reflected a DCR Total Score performance of 0–3, DCR Condition “Total 0–3, DCR Recall 0–2” reflected a DCR Total Score performance of 0–3 and a DCR Recall score of 0–2. Agreement rates were calculated as a proportion: the number of times a set of classifications were the same, divided by the total number of observations. Positive agreement is the agreement rate for the subset of participants for whom impairment is identified by the DCR, and negative agreement is the agreement rate for participants for whom impairment is not identified by the DCR. 95% CIs are based on the Agresti-Coull method for agreement rates

**Figure 1:**
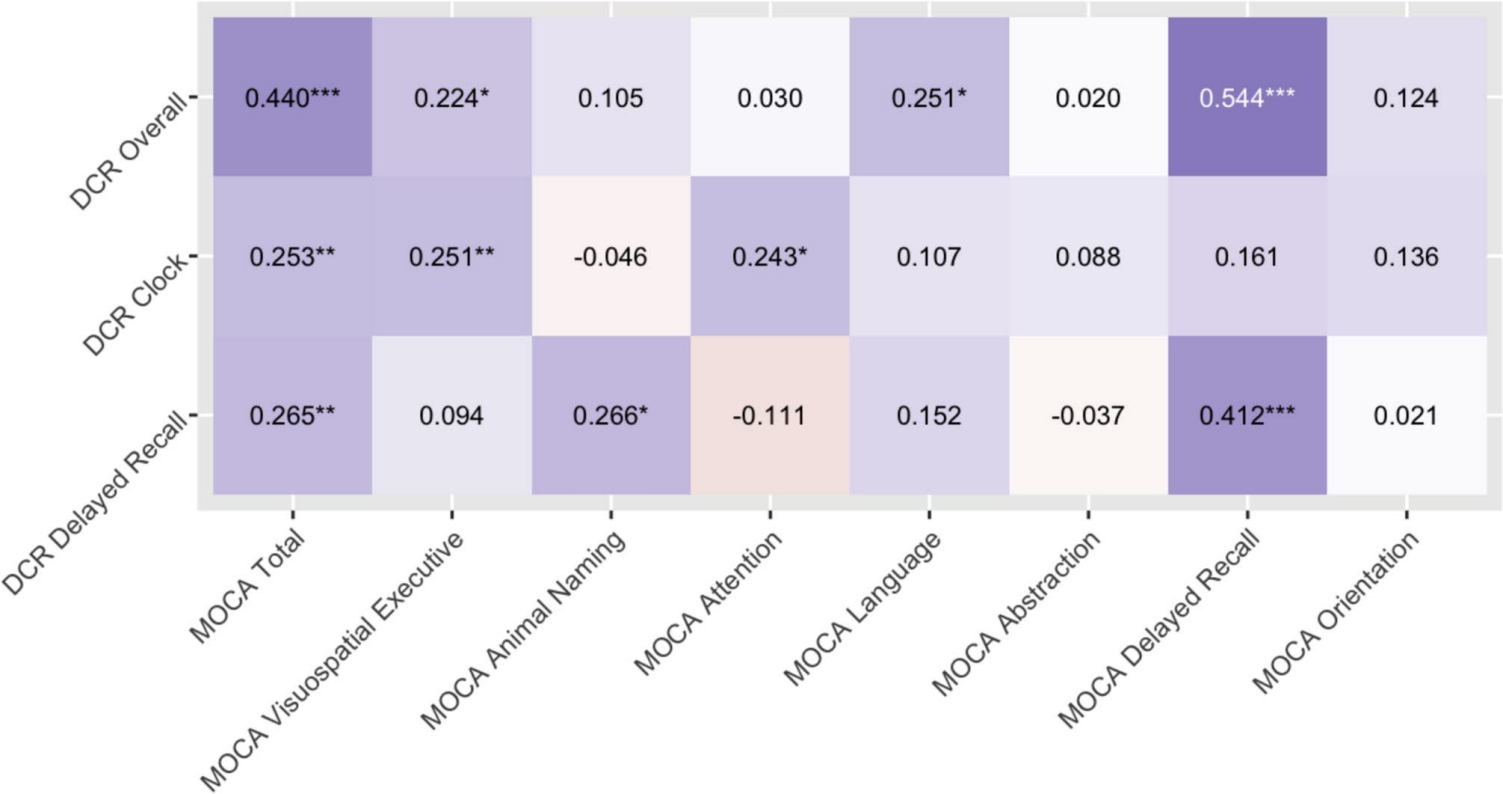
Correlation coefficients between DCR subtests and MOCA index scores. MOCA, Montreal Cognitive Assessment; DCR, Linus Health Digital Clock and Recall; DCTclock, digital clock drawing portion of the DCR. “*” denotes significant correlation, *p* < 0.05. “**” denotes significant correlation, *p* < 0.01. “***” denotes significant correlation, *p* < 0.001.

When examining convergent validity of the DCR, Fig. 1 indicates that the highest polychoric/polyserial correlations were between DCR Total Score and both MOCA Delayed Recall and MOCA Total Score (*p*s < 0.001), and between DCR Delayed Recall and MOCA Delayed Recall (*p* < 0.001). Smaller—but still significant—correlations were observed across several other relationships, including for DCTclock and DCR Delayed Recall subtests (*p*s < 0.01–0.05). No summary DCR scores were significantly correlated with MOCA indices of Abstraction or Orientation.

### Exploratory Data Analysis

Table 4 compares DCTclock composite subscales between groups stratified by MOCA Total Score impairment cutoff (Total Score < 26). Participants performing below MOCA impairment cutoffs possessed significantly lower DCTclock Spatial Reasoning scores for the copy version of the clock (Spatial Reasoning – Copy Clock; *p* = 0.003, *A* = 0.692) than participants performing above MOCA impairment cutoffs. A similar trend was seen for Spatial Reasoning for the command version of the clock (Spatial Reasoning – Command Clock; *p* = 0.051, *A* = 0.624). No other DCTclock subscales displayed differences across groups.

**Table 4:** Mean Rank of DCTclock Composite Subscales, Stratified by MOCA Impairment

DCTclock Composite Subscale	MOCA unimpaired	MOCA impaired	*U*	*p*	*A*	95% CI
Drawing Efficiency (Command Clock)	0.0 (1.1)	0.0 (1.5)	1211.0	0.806	0.484	0.370–0.603
Drawing Efficiency (Copy Clock)	− 0.2 (1.6)	− 0.6 (1.7)	1097.0	0.618	0.533	0.400–0.653
Simple/Complex Motor (Command Clock)	− 0.8 (1.8)	− 0.9 (1.4)	1224.0	0.736	0.479	0.351–0.614
Simple/Complex Motor (Copy Clock)	− 0.7 (1.8)	− 0.6 (1.1)	1182.0	0.950	0.497	0.360–0.634
Information Processing (Command Clock)	− 0.1 (1.4)	0.1 (1.9)	1090.5	0.592	0.536	0.417–0.655
Information Processing (Copy Clock)	− 0.1 (1.6)	− 0.4 (1.6)	1044.5	0.403	0.555	0.429–0.675
Spatial Reasoning (Command Clock)	− 1.5 (2.2)	− 2.0 (3.1)	883.0	0.051	0.624	0.512–0.729
Spatial Reasoning (Copy Clock)	− 0.7 (1.5)	− 1.7 (1.4)	723.0	0.003	0.692	0.554–0.801

*DCTclock*, digital clock drawing portion of the Linus Health Digital Clock and Recall Test; *MOCA*, Montreal Cognitive Assessment; *CI*, confidence interval; MOCA impairment cutoff set at Total Score < 26. Statistics represent the Mann–Whitney* U* and *p* values when comparing mean ranks of variables between groups stratified by MOCA impairment, along with associated Vargha and Delaney’s* A* effect sizes and 95% CIs

**Figure 2:**
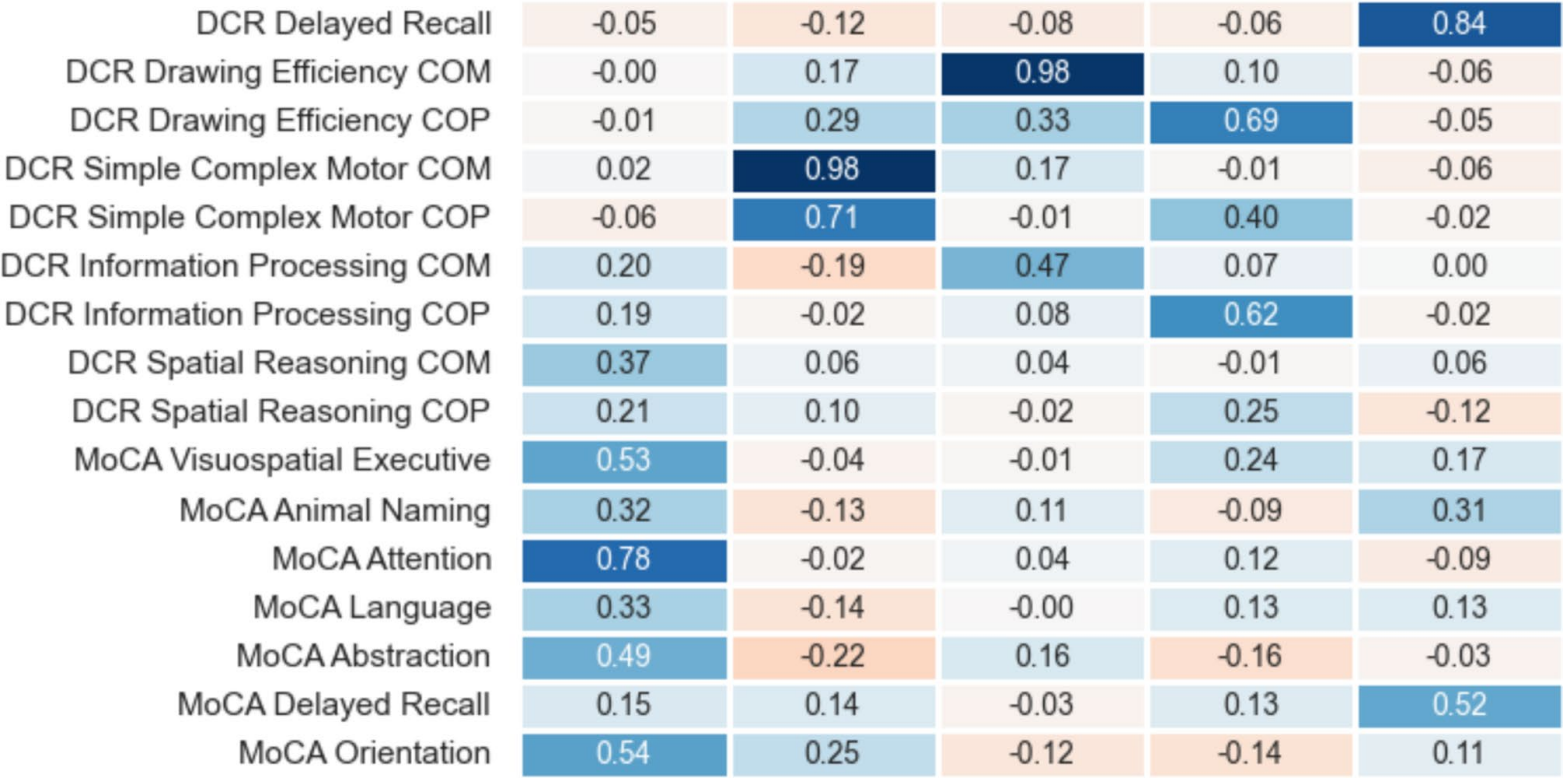
Factor loadings of exploratory factor analysis of MOCA and DCR components, indicating 5-factor model, with varimax rotation. DCR, Linus Health Digital Clock and Recall; COM, command subtest of clock drawing; COP, copy subtest of clock drawing; MOCA, Montreal Cognitive Assessment.

Finally, factor analysis showed that a five-factor model (orthogonal factor rotation [varimax]) was the simplest model that gave adequate data fit, as four-factor models failed to explain significant variance in the delayed recall items. Starting on the left of Fig. 2, the five factors loaded predominantly on the following: (1) on MOCA subscores along with loadings on Information Processing and Spatial Reasoning composite subscales of DCTclock; (2) on Simple and Complex Motor composite subscales of DCTclock; (3 and 4) on Drawing Efficiency and Information Processing composite subscales of DCTclock, with one factor focusing more on the command clock and another more on the copy clock; and (5) on delayed recall items in both DCR and MOCA.

## DISCUSSION

Current results highlight criterion and convergent validity of the Linus Health DCR in real-world primary care settings, relative to a “gold standard” MOCA cognitive screening tool. Consistent with hypotheses, DCR Total Score and select process scores successfully discriminated impairment on the MOCA using traditional cutoffs (Total Score < 26) in a sample of 114 primary care patients (Table 2). Given the novelty of this study, there is no previous literature to directly compare the validity between these two screening tools. However, these findings correspond with expectations given that the DCR is digital evolution of the Mini-Cog, which has shown reasonable diagnostic accuracy to detect cognitive impairment in primary care and specialty-MCI samples.^19^ Our results similarly converge with previous research suggesting greater diagnostic accuracy of the DCR relative to both Mini-Cog and MMSE for MCI/early dementia.^7,8^ One explanation for this observed validity is that both DCR and MOCA tap into multiple cognitive domains, in addition to serving as proxies for overall cognitive status. This latter aspect is desirable in a screening tool aimed at detecting cognitive impairment. Overall, our work provides supportive evidence for criterion validity for the DCR Total Score in primary care settings.

The DCTclock subscore appears to also be uniquely discriminatory of MOCA impairment. Regardless of the method used to score the clock, worse DCTclock performance was seen in those primary care patients classified as being impaired on the MOCA (Table 2). Compared to the others, the AI-based scoring algorithm displayed a slightly larger—but non-significant—effect than either the more simplified 0–2 point method or using the MOCA scoring rules. This is consistent with prior research finding the DCTclock algorithms to be superior to analog methods for detecting cognitive impairment.^20^ These results coincide with previous findings that the DCTclock successfully discriminated cognitively normal participants from those with MCI/early dementia and discerned amyloid status in cognitively intact participants.^21^ In particular, our exploratory analyses suggested that of the DCTclock composite subscales, Spatial Reasoning performed best after 95% CI comparison (Table 4). These results are consistent with literature showing that the Spatial Reasoning composite subscales displayed the greatest correlations with cerebral amyloid-PET and entorhinal tau-PET in participants along the AD continuum.^21^ Specifically, it has been proposed that the spatial arrangement of numbers and placement of hands on the clock face were significant drivers of this subcomponent’s association with AD pathology.^21^ Future research is warranted to better understand how such process-related scores enhance our understanding of early cognitive changes during neurodegeneration.

Evidence of convergent validity was observed for the DCR. Agreement rates of cognitive impairment using a variety of DCR and MOCA cutoffs were adequate, and concordance was best for cutoffs derived from Total Scores. Conversely, Table 3 reveals that incorporating DCR impairment cutoffs that require perfect performance on delayed recall led to low negative agreement (0.19–0.30) with those cases deemed to be cognitively intact on the MOCA (using either MOCA Total Score or Delayed Recall < 5). This surprisingly low negative agreement is likely because these analyses defined participants as being “cognitively intact” based on different cognitive profiles between the DCR and MOCA. In the second row of Table 3, “intact” participants needed perfect word-list performance on the DCR, but they could miss up to four items on the word-list on the MOCA. In the third row, “intact” participants needed good clock drawing and perfect word-list performance on the DCR, but they could miss any number of questions on the MOCA pertaining to executive, visuospatial, attention, or language skills. Although the positive agreement between these various subtest-based DCR and MOCA cutoffs was good (0.72–0.81) and cognitive screening tools often emphasize positive agreement (i.e., sensitivity), future examination of specificity of DCR component scores is recommended in larger samples to better understand their clinical utility.

Similarly, the DCR Total Score was significantly and positively correlated with the MOCA Total Score (*r*^2^ = 0.20), as well as with MOCA Delayed Memory, MOCA Language, and MOCA Visuospatial/Executive indexes (*r*^2^s = 0.05–0.30). Smaller magnitudes of association were observed for DCR subscales. Further, factor analytic results (Fig. 2) suggest that of the five factors generated from the various screening-measure subcomponents, one was comprised of memory subtests from both the DCR and MOCA, and another was comprised of non-memory MOCA subtests and Information Processing/Spatial Reasoning subscales of the DCR. Taken together, both DCTclock and Delayed Recall subtests appear to be related to their MOCA counterparts, which offers construct validity for the DCR subtests. However, the DCR Total Score displayed higher correspondence with MOCA than any individual subtest. This is likely due to both construct-related and psychometric phenomena, as summary scores tend to reflect more diverse cognitive abilities and wider ranges of scores. Consequently, although convergent validity appears to be observed across DCR and MOCA subcomponents, the greatest clinical utility for the DCR may lie in consideration of the DCR Total Score.

The current study is not without limitations. First, the current sample size was modest, which impacted our ability to observe significance for some analyses—especially those involving DCR and MOCA subcomponents. Future research on the DCR with larger samples of primary care patients is encouraged. Second, analyses did not adjust for demographically relevant covariates because the relative paucity of information on DCR performance led to this study being preliminary in nature. Third, the pragmatic design of the parent study meant that the current sample was comprised of only primary care patients who performed either as *Impaired* or *Borderline* on the DCR Total Score, as patients scoring 4–5 on the DCR Total Score were not referred for subsequent MOCA testing. As such, these results may not generalize to patients who perform intactly on the DCR. Relatedly, this resultant selection bias led to the analyses using positive/negative agreement instead of Kappa statistics. As a result of the restricted range of DCR Total Scores currently, correlations observed between DCR and MOCA comparisons were inherently constrained, which likely under-estimated the effect sizes for all other comparisons relative to variables possessing a full spectrum of performance. Studies examining a wider range of cognitive performance on both the DCR and MOCA are encouraged to avoid such restrictions to power and optimally compare these two screening tools in the future. Fourth, as the primary care settings were associated with Family Medicine—as compared to a specialty setting like Geriatrics—the range of cognitive severity observed in our sample was likely limited. Consequently, these findings should be replicated in more severe cognitively impaired samples to ensure that the validation holds across the spectrum of cognitive severity. Finally, although digital cognitive tools like the DCR possess many benefits, including the absence of a need for specialty-trained technicians^1^ and ease of incorporation into the electronic medical record,^22^ they introduce additional obstacles. These include questions pertaining to participant engagement and test validity,^23^ potential data security issues,^24^ and the need for both patients and clinics to have both a level of technological savvy^25^ and the technology available. Particularly in the latter case, the need for computing resources (e.g., tablets) and stable internet connections may render the use of digital assessment tools challenging in under-served or under-resourced regions/clinics. The interested reader is encouraged to review the clinical-decision tree created by Bloch and colleagues for a more thorough consideration of factors impacting the decision to implement remote tools for a particular patient.^26^

Despite these limitations, the current study provides evidence for the validity of the DCR as a cognitive screening tool in primary care settings. Given its streamlined and abbreviated administration time and reduced reliance on specialty-trained cognitive assessors, these results are promising and suggest that the DCR may have certain advantages over longer and more resource-demanding screening tools. Like any abbreviated cognitive task, the DCR is not currently intended to be a stand-alone tool for the diagnosis of MCI, but rather these results suggest utility as a front-line screener for cognitive impairment—particularly when focusing on the DCR Total Score as the variable of interest. The development of such easily incorporated tools into primary care workflows is capable of increasing the reach of cognitive screening across the general population. Consequently, when paired with parallel advancements in the detection of plasma-based biomarkers^27^ and recent FDA approvals for disease-modifying treatments for AD, the DCR and similar digital cognitive assessment tools have the potential to expand access to both AD diagnostic workups and subsequent treatments beyond specialty care.

## Data Availability

Data sharing for this project is available upon request and review by the Data Sharing Committee.
